# Exploring prehospital perceptions of interprofessional knowledge as a factor in emergency centre handover: a qualitative study of prehospital emergency care personnel

**DOI:** 10.11604/pamj.2026.54.3.42529

**Published:** 2026-05-06

**Authors:** Andrew William Makkink, Christopher Owen Alexander Stein

**Affiliations:** 1 Department of Emergency Medical Care, University of Johannesburg, Johannesburg, South Africa

**Keywords:** Patient handover, patient safety, paramedic, emergency department, interprofessional knowledge

## Abstract

**Introduction:**

a significant proportion of medical errors can be attributed to miscommunication between healthcare professionals. Effective interprofessional handover is heavily dependent on interprofessional collaboration, which can itself be affected by factors such as hierarchies, interprofessional differences, and unawareness of the other professionals' skillsets and expertise. The purpose of this study was to establish the potential effect(s) that interprofessional knowledge or lack thereof had on interprofessional communication and collaboration within prehospital to emergency centre (EC) patient handover.

**Methods:**

a qualitative descriptive design was used, with face-to-face, semi-structured interviews to gather data from 15 purposefully selected prehospital emergency care providers. Interviews were transcribed and imported into ATLAS.ti where they were coded using the principles of qualitative description. Data collection was concluded when saturation had been reached.

**Results:**

four dominant themes were identified relating to interprofessional knowledge as a factor in prehospital to emergency centre (EC) handover. These themes were: 1) lack of interprofessional knowledge, 2) lack of understanding of working environments, 3) prehospital understanding of pressures within the EC, and 4) the need for interprofessional education and collaboration.

**Conclusion:**

this is one of the few studies in a middle-income country relating the prehospital to the emergency centre patient handover. Improving interprofessional knowledge regarding qualification, capabilities, and working environment, interprofessional respect and communication can potentially improve interprofessional patient handover and patient safety. The findings of this study could be used to inform healthcare providers, policymakers, healthcare staff, and researchers to formulate intervention strategies to improve prehospital to emergency centre patient handover.

## Introduction

Up to 80% of medical errors involved some form of miscommunication between healthcare practitioners during the transfer of patients as estimated by the Joint Commission as far back as 2010 [1]. Patient handover within the South African emergency centre (EC) has previously been defined as “a patient-centred process that presents adequate and contextually relevant patient-specific information from one medical professional to another. Handover information is presented in a structured format that facilitates optimal information transfer and recall, as well as establishing a shared understanding of the patient´s condition, to ensure ongoing continuity of care. Handover serves to transfer responsibility and accountability for continuity of care from one medical professional to another. The handover process is complete once the receiving medical professional indicates (verbally or in writing) that they have taken over responsibility for the patient” [2]. The EC has been described as loud (with lots of noise such as screaming, shouting and the use of obscene language), chaotic, complex, fluid and one of busyness where time and resource constraints can impact communication effectiveness [3-6]. When compared to the rest of the hospital, the EC has a higher patient turnover, an unpredictable patient flow and a greater number of patient interventions per patient, per unit of time [7,8]. There is evidence to suggest that the process of patient handover at the prehospital to EC handover juncture is particularly vulnerable and that there is a risk that important information will be lost, the consequences of which may adversely affect patient well-being [9,10]. Effective patient handover within an environment such as the EC relies heavily on teamwork, interpersonal communication and interprofessional collaboration and it is therefore important to identify and mitigate potential barriers to effective handover. There is a paucity of literature relating directly to handover between paramedics and the EC.

Communication as a component of patient handover is of such importance that it has been claimed that for the quality of healthcare to improve, communication between interdisciplinary healthcare providers must improve [11]. Interprofessional communication (IPC) has been defined as “the sharing of information (by means of verbal, writing or other media) among members of different health professionals to influence patient care positively” [12]. When interprofessional communication fails, there are usually two predictable and distinct ways in which it fails, through silence and violence [13]. These failures manifest as important information being passed on to the wrong people, not being passed on at all, verbal attacks and sarcasm, and labelling or disqualification of other professionals [14]. Successful interprofessional communication is a critical aspect of interprofessional collaborative practice and teamwork [11,12,15].

Interprofessional collaboration can be defined as “multiple health workers from different professional backgrounds working together with patients, families, caregivers and communities to deliver the highest quality of care” [16]. Several factors have the potential to affect interprofessional handover, including interprofessional differences, a lack of established relationships, hierarchical relationships, infrequent face-to-face communication and unawareness of the other professionals´ skillset and expertise [12,17,18]. Where IP collaboration is effective, the benefits include mitigation of established hierarchies, decreased inaccurate information being relayed, decreased patient adverse events with resultant decrease in hospital stay and cost, a reduction in duplicated work as well as a reduction in tension and conflict and decreased staff turnover [18-20]. There is evidence to suggest that the more team members know about each other and their roles, the more effective that team becomes [19,20]. The implication is that better interprofessional knowledge may affect factors such as communication and collaboration related to improved patient handover.

Ineffective handover carries with it risks to patient safety, one of which may be negative consequences related to interprofessional collaboration due to a lack of interprofessional knowledge. This study aimed to establish the potential effect(s) that interprofessional knowledge or lack thereof had on interprofessional communication and collaboration within emergency centre (EC) patient handover.

## Methods

**Study design:** a qualitative, descriptive design was used to gather data from purposefully selected participants in an emergency care setting using semi-structured, face-to-face interviews. To ensure comprehensiveness of reporting, the ‘consolidated criteria for reporting qualitative research' (COREQ) guidelines were followed [21].

**Study setting:** the study took place in South Africa, a country comprising nine provinces, including Gauteng. Gauteng is the smallest province by area but the most populous, with an estimated population of 15 million [22]. Data were collected in the City of Johannesburg, one of the three primary metropolitan areas within the Gauteng Province.

**Study population and sampling strategy:** the population was made up of all categories of registered prehospital emergency care personnel (PECP) within the study area. There were several prehospital qualifications unique to the South African environment, allowing for registration in a scope-dependent registration category with the Health Professions Council of South Africa (HPCSA) [23]. Within the HPCSA, the Professional Board for Emergency Care (PBEC) governs prehospital emergency care personnel (PECP), and each registration category is directly related to qualification and scope of practice. Generic terminology is often used to categorize these PECP: advanced, intermediate, or basic life support. Advanced life support commonly refers to emergency care practitioners (four-year tertiary qualification), national diplomates (three-year tertiary qualification), critical care assistants (9-month course), emergency care technicians (two-year course), and diplomates (2-year tertiary qualification). Intermediate life support is generally provided by ambulance emergency assistants (three-month course), and basic life support is generally provided by basic ambulance assistants (four-week course) [23]. The study population consisted of all categories of PECP who were working in the study area during data collection. Potential participants were approached at their places of work using a purposive, convenient recruitment strategy by verbally introducing the study and then providing them with a paper-based information document outlining the study. Interviews were conducted between October 2018 and March 2019. Persons who agreed to participate were then engaged to determine a time and venue for the interview if this was not immediately possible.

**Data collection and procedure for the interviews:** face-to-face, semi-structured interviews were conducted using the interview guide that focused on areas of interest identified during the quantitative phase of a mixed-methods study. The questions were structured around the assumption of the PECP being the deliverer of handover and either a doctor or a member of the nursing staff being the receiver. Two recording devices were used to record the interview. The pilot testing of the interview guide was carried out by discussing with two interviewees to determine whether they perceived any ambiguity or lack of clarity in the questions. There were no identified issues, and the interview guide was left as is. All interview venues were chosen by the interviewees and deemed to provide adequate privacy and confidentiality. Field notes were taken by the principal investigator (PI) during the interviews and were referred to during the analysis of the data where relevant.

**Ethical approval of the study and informed consent:** this study was conducted in accordance with the 964 Declaration of Helsinki and its later amendments or comparable ethical standards. Ethical approval for the study was granted for the initial project by the University of Cape Town´s Human Research Ethics Committee (624/2012) in December 2012 and was renewed annually for the duration of the study. Approval was sought and granted by the relevant employers in cases where participants were approached at their places of work. Informed consent was ensured by providing all interviewees with an information document to read through that specified the aims of the study as well as the voluntary nature of participation, confidentiality, and their right to withdraw from the study at any point. Interviewees were also required to sign a document confirming informed consent to be interviewed as well as consent to be audio-recorded prior to the interview commencing.

**Transcription of data:** the audio-recorded interviews were transcribed by a professional transcription company that signed a confidentiality agreement. The transcribed data was sent to the PI via a secure link and then checked for accuracy by the PI, and where relevant, corrections were made to the transcription. The transcriptions were then imported into ATLAS.ti (version 8, ATLAS.ti Scientific Software Development GmbH, Berlin, Germany) after which there was no further editing.

**Data analysis and rigor:** the data were analysed using qualitative content analysis, which is the analysis strategy of choice in qualitative descriptive studies [24]. Interviews were initially transcribed verbatim into a Microsoft Word® (Office 2016, Microsoft Corporation, Redmond, WA) document. A final grammar check of the transcriptions was performed by the PI, and where relevant, grammar was changed to accurately reflect medical terminology. Transcriptions were imported into ATLAS.ti for analysis. Computer-assisted qualitative data analysis software (CAQDAS) tools have been associated with more rigorous data analysis that is less time-consuming to perform and organise than traditional methods, which is why ATLAS.ti was employed [25,26]. Interview transcriptions were read both vertically and horizontally several times to ensure better immersion into the data [27]. Coding was carried out horizontally and vertically using a primarily open, inductive, and deductive strategy. Vertical coding involved assigning codes within single interview transcripts, and horizontal coding involved coding within question responses across the sample [28]. The sample size was guided by data saturation principles that included the depth and richness of the data, as opposed to the number of participants [29]. Data were collected and analysed until limited fresh insights were being revealed, and where adding more participants would not have resulted in new information being gained [30,31].

The consistency and validity of the coding framework were evaluated using a code/recode strategy. The method used was for the PI to perform a code-recode of the data on two separate occasions, and these coding lists and the interview transcripts were reviewed and compared by one of the co-investigators who had not been involved in the coding process. The formation of categories was carried out by structuring the data using both concept-driven and data-driven strategies. Using a code-recode strategy ensured dependability, as did comparing the results from the two rounds of coding for congruency. A high level of congruence was observed, and the use of a qualitative descriptive approach ensured authenticity by generating thick and rich verbatim quotes from the participants. This approach also demonstrated the emotions and feelings of the participants in their own words through the use of direct quotes.

Credibility was ensured by ensuring that a range of qualifications from the prehospital environment were interviewed and that a holistic perspective was captured and portrayed. By including all data from the interviews, we ensured that the full interviewee perspective was explored. The qualitative descriptive methodology that was used addressed confirmability by lending itself not so much to the interpretation of a perceived meaning but rather to presenting participant responses as they were.

The male PI (PhD) was an emergency care practitioner employed at a local university with over 20 years of experience in the prehospital environment and had been involved in several previous studies, both quantitative and qualitative, and had training in CAQDAS, specifically ATLAS.ti. The interest in the research topic had come from the PI´s own experience and interactions with healthcare practitioners who had indicated that handovers were a generally poor area within patient management and safety. Qualitative descriptive research is founded in existing knowledge, thoughtful linkages to the work of others, and the clinical experience of the researcher(s) [32]. Reflexivity is defined as “a set of continuous, collaborative, and multifaceted practices through which researchers self-consciously critique, appraise, and evaluate how their subjectivity and context influence the research processes” [33]. Within the qualitative descriptive paradigm, the PI used constant reflexivity to consider their subjectivity and bias as a paramedic within the context of the study, from the design of the interview schedule to the analysis and interpretation of the data. These biases were mitigated through an effort to remain neutral during interviews and constant reflection on bias during the data collection and analysis. The potential biases of the PI were further mitigated by a co-investigator who manually analyzed the codes for consistency and determined that there was adequate consistency between the two sets of codes as well as with their own interpretations of the coding tree. The PI´s motivation for the study and subjectivity were also shared with participants when deemed relevant.

## Results

A total of 15 face-to-face, semi-structured interviews were conducted with prehospital emergency care personnel who were selected using purposive sampling within the Johannesburg area in South Africa. The HPCSA registration category and experience demographics of the interviewees are depicted in Table 1. The participants consisted of seven emergency care practitioners with a mean of more than 12 years of experience, three critical care assistants with a mean of 23 years of experience, one emergency care technician with 18 years of experience, three ambulance emergency assistants with a mean of more than 15 years of experience, and one basic ambulance attendant with four years of experience.

**Table 1:** prehospital emergency care personnel participant qualification, experience, and interview length

Interviewee registration category	Interview number	Years qualified at current qualification (years)	Total prehospital experience (years)	Interview length
Emergency care practitioner	ECP01	3	23	17 min 39 sec
Emergency care practitioner	ECP02	4	10	28 min 15 sec
Emergency care practitioner	ECP03	3	7	21 min 29 sec
Emergency care practitioner	ECP04	3	15	14 min 38 sec
Emergency care practitioner	ECP05	2	9	21min 54 sec
Emergency care practitioner	ECP06	5	19	21 min 43 sec
Emergency care practitioner	ECP07	2	6	15 min 36 sec
Paramedic	CCA01	25	35	15 min 38 sec
Paramedic	CCA02	7	25	18 min 06 sec
Paramedic	CCA03	2	9	26 min 30 sec
Emergency care technician	ECT01	5	18	25 min 30 sec
Ambulance emergency assistant	ANA01	11	15	11 min 41 sec
Ambulance emergency assistant	ANA02	26	28	13 min 39 sec
Ambulance emergency assistant	ANA03	1	4	16 min 05 sec
Basic ambulance assistant	BAA01	5	4	14 min 46 sec

ECP: Emergency care practitioner; CCA: Critical care assistant; ECT: Emergency care technician; ANA: Ambulance emergency assistant; BAA: Basic ambulance assistant

**Themes identified:** four themes were identified in the data that related to perceptions that PECP had regarding interprofessional knowledge within the context of EC handover: 1) the perception that EC personnel did not have adequate knowledge nor respect related to prehospital qualification structures and scopes of practice, 2) the perception that EC personnel did not understand the prehospital working environment and the reciprocal was true, 3) the perception that PECP understood the pressure at play in the emergency centre, 4) the perception that better interprofessional education and interprofessional collaboration were potential solutions to improving handover between prehospital and the EC personnel.

**Theme 1: perceived lack of knowledge:** there was a perception amongst respondents that EC personnel did not have an adequate understanding of the prehospital qualifications and scopes of practice, and linked this to a lack of respect. “*I think rather they don't respect or recognize the education or the qualification, and I think it´s driven by the fact that they are ignorant of our scopes of practice, how we are educated, how we're trained for what we do*” (ECP02). “*And they don´t respect us because maybe they look down on our qualifications*” (CCA03). “*They don´t really understand what we do … I don´t think they know as well that the levels or qualifications. I think they don´t know what our ranking structure is or the capabilities of different EMS personnel. And that´s where sometimes you will get an ignorant attitude*” (ECT01). One participant mentioned that a similar problem was prevalent amongst PECP related to EC personnel qualifications and scope. “*And it goes both ways. I mean, when it comes to your nurses and stuff, I don´t know what everyone [does] ... I´ve got a vague idea, but you don´t always know what every nurse and every person can do specifically, so it goes both ways*” (ANA03).

**Theme 2: understanding of environment:** respondents expressed frustration related to the perception that EC personnel did not understand the challenges that were prevalent while working in the prehospital environment. “*They might have all the qualifications … and everything, but they have absolutely no idea what´s happening pre-hospital*” (ECP01). “*I mean, they don´t know the availability of back-up on the road or the situation, or the distance from the call to the hospital*” (ANA01). “*… they'll belittle you, and I think sometimes it actually has to do with the lack of understanding of the prehospital environment, you know*” (ECP07). Respondents also expressed a perception that PECP were more exposed to the EC environment in their training than EC personnel were to the prehospital environment, and that EC personnel who had been exposed to the prehospital environment were more understanding. “*I do feel that the pre-hospital training is far more committed to exposing their students or their learners to the in-hospital environment than the in-hospital environment is committed to exposing their learners to the pre-hospital environment… I think we have a better understanding of them than they do of us, purely because our training forces and requires that of us*” (ECP02). “*… they come to the pre-hospital environment as well to get exposure and the likes in terms of the environment that we are working in… they´ve got an understanding and a positive outcome of the pressure that we are working under and the likes*” (CCA03).

**Theme 3: resources in the EC:** respondents acknowledged that there was an understanding of the pressures at play in the EC and acknowledged that ECs were generally busy and understaffed. “*… sometimes they are just overworked, and they´re just, they´re just run off their feet, overworked, understaffed …*” (ECP01). “*…they are inundated with all the other responsibilities, particularly administrative and patient movement…*” (ECP02). “*… I think either they are understaffed or the hospitals are overworked or they´re over, they´ve got too many patients during the day…*” (ECT01). One respondent indicated that prehospital personnel needed to spend more time understanding the EC environment. “*I think the pre-hospital guys should spend more time understanding the pressures the emergency staff are under. This expectation, especially with the non-critical patients, to walk over and hand over in five minutes and get out, I think, is unfair*” (ECP02).

**Theme 4: interprofessional education and interprofessional collaboration:** respondents suggested several strategies for IPE and IPC. These included that cross-pollination and working in each other´s environments would be helpful, but also that any intervention should be reciprocal. “*I think that maybe it might be a good idea to have some type of cross-pollination of having the emergency centre staff working on the road and vice versa, us in the emergency centers*” (ECP06). “*I think you know it would be helpful if they worked some pre-hospital shifts with us and experienced what we experience on the road. At the same time, I think it would be helpful if it were the other way around, if we perhaps worked in more situations that they are involved in*” (ECP07). “*… if they had a better understanding of what our skills and our scope of practice were, I honestly believe the handovers would change dramatically*” (CCA02). “*So it is very, very important so that we can get the education. I think we are not trained enough…*” (BAA01). Interestingly, one respondent mentioned that there may be an unwillingness to work or learn in each other´s environments. “*… you know, you can take them out of their environment and put them into our environment, but they still won't learn because they want to be in their environment, and the same with us. You can put us in their environment, and we'll just think, you know, this is not for us*” (ECP03).

## Discussion

Interprofessional knowledge was perceived as a factor affecting the efficacy of EC handover between the PECP and the EC personnel. This perception relating to interprofessional knowledge is supported by Hallikainen *et al*. who advocated that “it is also important for physicians to understand the work of paramedics, and vice versa” [34]. However, the perception of respondents in our study that EC personnel had a poor knowledge of PECP´s training, clinical abilities, and scopes of practice seems to confirm the findings in the study by Vincent-Lambert *et al*. [35]. In this South African study, 64% of soon-to-graduate doctors, nurses, and clinical associates demonstrated a poor understanding and 36% a moderate understanding of the training and clinical abilities of emergency care practitioners [35]. There was a perception amongst PECP in this study that EC personnel had a poor knowledge of the prehospital qualifications, and there are several potential reasons for this. The historical context of prehospital qualifications in South Africa and the recent changes in qualification structure implemented by the HPCSA may be a factor related to EC personnel being perceived as having limited knowledge related to prehospital qualifications and clinical capabilities. In 2015, van Vleet described four major hurdles for paramedic transition into a profession, and these are not dissimilar to the path taken by the South African prehospital emergency care profession. The four major hurdles are: shifting from vocational to baccalaureate/graduate education, transitioning into an acknowledged profession, expansion of role and scope of practice, and extending clinical oversight of care [36].

Furthermore, this shift from vocational to graduate education in South Africa created a multi-levelled system where some of the traditional vocational qualifications have been removed from the qualification mix and new graduate qualifications added [23]. This change has resulted in a wide array of qualifications and related scopes of practice that are not easy to compartmentalise nor to identify, and has further been compounded by the shift to evidence-based practice by the publication of the Clinical Practice Guidelines (CPG). The CPGs have affected the relevant expansion and, in some cases, reduction of the scopes of practice and clinical capabilities [37]. The CPGs caused considerable uncertainty within the prehospital emergency care profession, with people being unsure of where the profession was heading [38]. Given this context, it is not surprising that EC personnel are perceived as having limited knowledge related to PECP qualifications and clinical capabilities. The same may be true for interprofessional knowledge of PECP in other countries when the capabilities of practitioners are revised, restructured, or remain unclear [39]. Despite this, evidence exists encouraging nurses and doctors to learn more about PECP and to learn to trust their patient management [40]. It is becoming more critical for IPE and IPC strategies to be implemented so as to ensure improved interprofessional knowledge.

Respondents indicated that EC personnel did not understand the environment and challenges that PECP faced in the prehospital environment, whereas they felt that they were aware of the challenges faced by EC personnel, such as overworking and understaffing. Literature is scarce regarding EC staff perceptions of the prehospital working environment, even though many nurses and doctors have, at some time or another, worked with paramedics. Emergency nurses working in the SA prehospital environment have indicated that the prehospital environment is difficult and unpredictable, with extended transport times [41]. The caveat is that for someone to understand this, they need to work in the prehospital environment, something which does not seem to be common practice amongst EC staff. Where PECPs have the perception that their qualification or working environment is not respected, this has the potential to breed disrespect, which quickly may become mutual. Poor interprofessional respect may often manifest within the dynamics of identification processes, where different professions or persons start to identify themselves as being in opposition to others.

According to Pedersen *et al*. this opposition can potentially create an “us-them relationship” and if the conflicts remain unresolved, communication, collaboration, teamwork and, ultimately, patient safety can be negatively affected [42]. This opposing mindset is contrasted with improved interprofessional collaboration when people feel part of a team [43]. Working with paramedics in the prehospital environment has been shown to give emergency nurses a better understanding of the working environment and relevant challenges faced by PECP as well as the importance of team dynamics and that improved knowledge and mutual respect between the various role players had a positive effect on delivery of patient care [41]. Strategies should therefore be explored aimed at improving interprofessional knowledge, specifically related to qualifications, scope and working environment, with the goal of improving interprofessional communication, collaboration, and teamwork.

There was broad agreement amongst participants regarding the perceived significant need for education and training as a strategy to improve interprofessional knowledge. Interprofessional education should not merely be a paper- or classroom-based activity, there should be a focus on co-learning. In line with the recommendations of the Centre for the Advancement of Interprofessional Education (CAIPE), interprofessional education and training should be “when two or more professionals learn with, from and about each other to improve collaboration and the quality of care” [44]. The sentiments of IPE and structured IPC are echoed in several other studies, some of which mention that people just need to get to know each other as a start [35,45]. Similarly, co-education in emergency medicine of medical and paramedic students has shown value within purposeful professional medical education [34]. Trans-professional mentoring, as identified by participants in this study, has also been suggested to improve interprofessional knowledge and understanding of roles [12].

In addition, when designing any interprofessional educational intervention, it is important to remain cognisant of the two primary discourses within interprofessional education, namely the utilitarian and the emancipatory discourses. The utilitarian discourse requires evidence and validity for successful outcomes for those receiving care, and the emancipatory discourse relates to relationships, including those of power and dominance, between practitioners [44,46]. These interventions should be implemented as early as possible, and there is increasing evidence to suggest that pre-qualification learners exposed to IPE and IPC are demonstrating increasingly positive outcomes that include better attitudes towards one another and gains in knowledge and skills required for collaborative practice [47]. Literature highlights the benefits of IPE and IPC between PECP and the EC, and strategies must be implemented to improve these and in doing so, patient safety [48,49].

**Figure 1:**
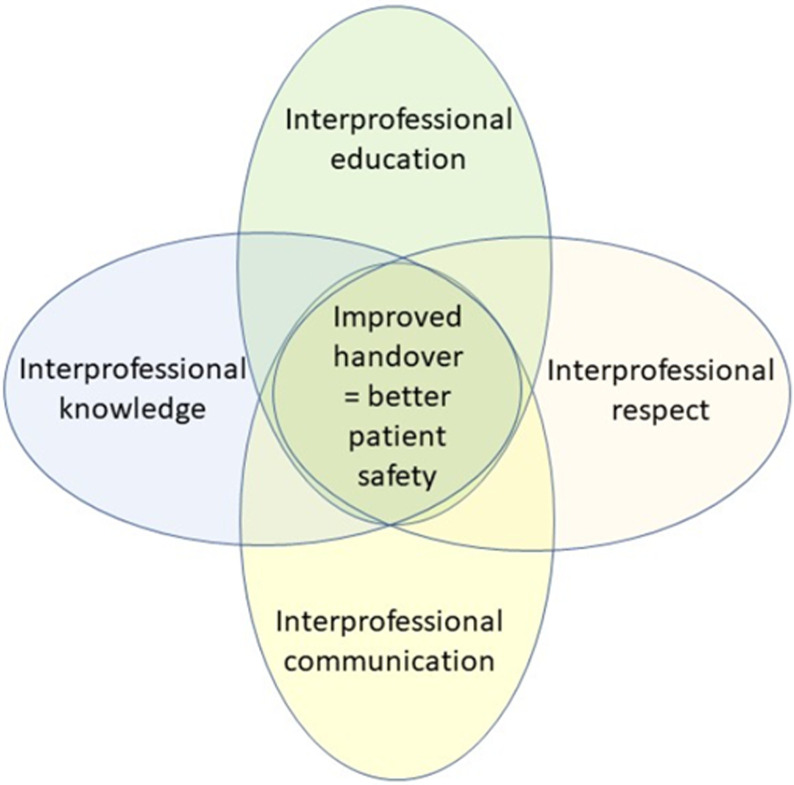
a relational diagram depicting how principles of interprofessionality could be linked to improved EC handover

Based on the findings of this study, we propose a relational diagram, depicted in Figure 1, that encapsulates how the four identified principles of inter-professionality (interprofessional knowledge, respect, communication, and education) have the potential to affect EC handover. The four aspects identified were interprofessional knowledge that had links to interprofessional communication and respect, and to interprofessional education (or lack thereof). Addressing all four of these would ultimately result in the potential for improved EC handover and improved patient safety.

**Limitations:** the primary limitation of this study was that it explored only the prehospital (deliverer) perspective of EC handover. Further limitations were that it was geographically limited to one urban area, and although we interviewed a relatively small sample, we included a range of qualifications and achieved data saturation during analysis. Researcher biases are a limitation of any qualitative study, but we initiated several mitigation strategies as described to minimize these. The results may not be generalizable to all EC handovers or professionals who interact within the healthcare system, yet they provide useful principles to guide strategies aimed at improving EC handover.

## Conclusion

This study explored PECP perceptions of interprofessional knowledge as a factor in EC handover. Prehospital emergency care personnel perceived a lack of adequate understanding amongst EC staff related to the knowledge and scope of prehospital qualifications and scopes of practice. Prehospital emergency care personnel expressed frustration regarding the perceived lack of knowledge regarding the prehospital working environment, but indicated that they understood the pressures at play in the EC. There was a strong theme regarding the need for IPE and IPC. Improving interprofessional knowledge regarding qualification, capabilities, and working environment, interprofessional respect, and communication can potentially improve interprofessional patient handover and patient safety. The findings of this study could be used to inform healthcare providers, policymakers, healthcare staff, and researchers to formulate intervention strategies to improve prehospital to emergency center patient handover. Interprofessional education and collaboration strategies aimed at improving patient handover should be implemented in both prehospital and in-hospital settings. Further research should be conducted focusing on the EC perspective. In addition, the potential structure and content of education and training programs should be investigated, as well as how best to facilitate professionals learning with, from, and about each other.

### What is known about this topic

- Interprofessional handover remains a period of vulnerability and risk within the patient care continuum;
- Effective interprofessional communication, collaboration and teamwork contribute to the patient receiving the highest quality of care.

### What this study adds

- A lack of interprofessional knowledge and understanding of working environments and scopes between prehospital and EC staff can negatively affect handover;
- Improving interprofessional knowledge, interprofessional respect and communication can potentially improve interprofessional patient handover and patient safety;
- The findings of this study could be used to inform healthcare providers, policymakers, healthcare staff, and researchers to formulate intervention strategies to improve prehospital to emergency centre patient handover, as well as handovers in other areas of the patient care continuum.
